# Phenotype-Specific Mortality Outcomes with Dipeptidyl Peptidase-4 Inhibitors in Heart Failure and Diabetes: Real-World Evidence from a Retrospective Single-Center Cohort Study

**DOI:** 10.3390/jcm15083153

**Published:** 2026-04-21

**Authors:** Lama Alfehaid, Ahmad Alamer, Atheer Alhantush, Majed S. Al Yami

**Affiliations:** 1 Department of Pharmacy Practice, College of Pharmacy, King Saud bin Abdulaziz University for Health Sciences, Riyadh 11481, Saudi Arabia; yamim@ksau-hs.edu.sa; 2 Pharmaceutical Care Department, King Abdulaziz Medical City, Riyadh 11426, Saudi Arabia; ath16hn@gmail.com; 3 King Abdullah International Medical Research Center, Riyadh 11426, Saudi Arabia; 4 Department of Clinical Pharmacy, Prince Sattam bin Abdulaziz University, Al-Kharj 11942, Saudi Arabia; aa.alamer@psau.edu.sa

**Keywords:** heart failure, type 2 diabetes mellitus, dipeptidyl peptidase-4 inhibitors, sitagliptin, cardiovascular mortality, heart failure phenotypes, real-world evidence, propensity score overlap weighting

## Abstract

**Background/Objectives**: Type 2 diabetes mellitus (DM) commonly coexists with heart failure (HF) and is associated with increased cardiovascular (CV) morbidity and mortality. Although dipeptidyl peptidase-4 (DPP-4) inhibitors are widely used for glycemic control, their CV safety in patients with established HF, particularly across HF phenotypes, remains uncertain. To evaluate the association between DPP-4 inhibitor use and 6-month CV mortality in patients with DM and HF, and to assess whether this association differs across HF phenotypes: HF with preserved ejection fraction (HFpEF), HF with mildly reduced ejection fraction (HFmrEF), HF with reduced ejection fraction (HFrEF). **Methods**: We conducted a retrospective cohort study at King Abdulaziz Medical City from January 2017 to December 2024 that included adults with DM and echocardiographically confirmed HF. Patients receiving DPP-4 inhibitors were compared with non-users. The primary outcome was 6-month CV mortality. Propensity score overlap weighting targeting the average treatment effect in the overlap population was applied to balance baseline characteristics. Weighted logistic regression with interaction terms was used to assess effect modification by HF phenotype. **Results**: Among 3435 patients (median age 69 years; 51.3% female), 1921 (55.9%) received a DPP-4 inhibitor, predominantly sitagliptin. In unadjusted analyses, CV mortality was numerically lower among DPP-4 inhibitor users across HF phenotypes. However, after overlap weighting, CV mortality was similar between users and non-users within HFpEF (7.1% vs. 8.0%; OR 0.88, 95% CI 0.51–1.52; *p* = 0.646), HFmrEF (2.6% vs. 5.0%; OR 0.50, 95% CI 0.09–2.86; *p* = 0.436), and HFrEF (6.4% vs. 6.4%; OR 1.00, 95% CI 0.48–2.07; *p* = 0.992). No significant interaction was observed between DPP-4 inhibitor use and HF phenotype (interaction *p* > 0.05). **Conclusions**: In this large real-world cohort of patients with DM and established HF, DPP-4 inhibitor use was not associated with increased or reduced 6-month CV mortality after robust adjustment. The neutral association was consistent across HF phenotypes, supporting the CV safety of DPP-4 inhibitors, predominantly sitagliptin, in contemporary HF management.

## 1. Introduction

Diabetes mellitus (DM) and heart failure (HF) are two highly prevalent chronic conditions that impose a major global health burden [1]. Their relationship is bidirectional: DM increases the risk of developing HF, while HF itself has been shown to raise the likelihood of new-onset DM [2,3,4]. The Framingham study and subsequent population-based analyses demonstrated that patients with DM have a two-to fivefold higher risk of cardiovascular (CV) disease, including HF, independent of other atherosclerotic conditions [3]. Shared mechanisms such as hyperglycemia, insulin resistance, neurohormonal dysregulation, lipotoxicity, and systemic inflammation contribute to the development and progression of both diseases [5]. Understanding these interactions is crucial to inform therapeutic strategies that target common pathways.

The CV burden is particularly evident in HF, one of the fastest-growing CV disorders and a leading cause of mortality worldwide [6]. Effective glycemic control is therefore essential, not only to mitigate DM complications but also to reduce HF incidence [7]. Landmark trials have established robust CV benefits for sodium–glucose cotransporter 2 (SGLT2) inhibitors and glucagon-like peptide-1 receptor agonists (GLP-1 RAs) [8,9]. In contrast, the role of dipeptidyl peptidase-4 (DPP-4) inhibitors remains controversial. These oral agents are widely prescribed due to their safety profile and ability to reduce HbA1c, and they exert additional pleiotropic effects on the CV and renal systems [10,11,12].

Evidence from large, randomized trials has been mixed. Sitagliptin (TECOS) and linagliptin (CARMELINA) demonstrated CV safety, with no increase in the risk of HF hospitalizations [13,14]. Conversely, saxagliptin (SAVOR-TIMI) and alogliptin were associated with a higher risk of HF hospitalization despite noninferiority in major adverse CV events [15,16]. Real-world data also suggest higher HF event rates with DPP-4 inhibitors compared with SGLT2 inhibitors [17]. Importantly, no trial has shown a mortality benefit in patients with HF, and data on specific HF phenotypes (HFrEF vs. HFpEF) remain limited [18,19].

Although several global studies have evaluated the cardiovascular safety of DPP-4 inhibitors, evidence regarding their impact on HF outcomes remains inconsistent, particularly in real-world populations with a high burden of cardiometabolic comorbidities. Most available data are derived from randomized trials or administrative databases that may underrepresent patients with advanced disease, multimorbidity, or prolonged follow-up. Addressing this gap is clinically important, as DM and HF frequently coexist and continue to rise worldwide, creating uncertainty regarding optimal glucose-lowering strategies in patients with established or evolving heart failure. Generating real-world evidence in diverse healthcare settings can help inform clinical decision-making and improve risk stratification in this growing high-risk population.

## 2. Materials and Methods

### 2.1. Study Aim and Objectives

The primary objective of this study was to evaluate the association between DPP-4 inhibitor use and cardiovascular-related mortality in patients with type 2 DM and HF. Secondary objectives included comparing outcomes across HF phenotypes, HFrEF vs. HFpEF.

### 2.2. Study Design and Setting

This was a retrospective observational study conducted at King Abdulaziz Medical City, Ministry of National Guard Health Affairs (MNGHA), Riyadh, Saudi Arabia. Data were extracted from the institution’s electronic health records system (BestCare) over the study period from 1 January 2017 to 31 December 2024.

### 2.3. Study Population

Eligible participants were adults (≥18 years) with an established diagnosis of DM and HF, confirmed by echocardiography, who had received at least one prescription for a DPP-4 inhibitor during the study period. Patients were excluded if they lacked an established HF diagnosis or had incomplete clinical records for at least 6 months following initiation of DPP-4 inhibitor therapy.

DPP-4 inhibitor exposure was defined at baseline based on the presence of an active prescription at cohort entry (index date). Patients were classified according to their baseline treatment status and followed for a fixed period of six months. Exposure was analyzed using an intention-to-treat framework, whereby patients were analyzed according to their baseline treatment assignment regardless of subsequent treatment changes during follow-up.

### 2.4. Sample Size and Sampling Technique

Given the retrospective design, all eligible patients were included, and no formal a priori sample size calculation for cardiovascular mortality was performed. The analysis was exploratory in nature and intended to provide real-world estimates across HF phenotypes.

### 2.5. Outcome Definition and Ascertainment

The primary outcome was CV mortality within six months of cohort entry. Cardiovascular death was defined based on the primary cause-of-death classification recorded in the institutional electronic health record system (BestCare).

CV mortality included deaths attributed to acute myocardial infarction, heart failure, stroke, sudden cardiac death, fatal arrhythmia, or other documented cardiovascular causes.

Cause-of-death information was extracted from structured diagnosis fields and verified through review of hospitalization records, discharge summaries, and death documentation when available.

### 2.6. Data Management and Analysis

Data were extracted from the electronic health records system (BestCare) at King Abdulaziz Medical City, Riyadh, using a standardized electronic case report form. A secure, password-protected database was created to store and manage study variables. Each patient was assigned a unique study identifier to maintain confidentiality, and all personal identifiers were removed prior to analysis. Access to the dataset was restricted to study investigators. Data quality was ensured through logic checks for inconsistencies, cross-referencing with original charts as needed, and double-entry verification of a random subset of records.

Baseline characteristics were summarized using medians with interquartile ranges (IQR) for continuous variables and counts with percentages for categorical variables. The Mann–Whitney U test was used to test two-group comparisons, and the Kruskal–Wallis test for comparisons across multiple heart-failure subtypes. Chi-square tests were applied for categorical variables. Missing baseline data were imputed using multiple imputations via chained equations (MICE) under the assumption of missing at random (MAR) [20]. One imputed dataset was selected for reporting descriptive characteristics and covariate balance diagnostics.

To minimize confounding due to treatment selection, we applied propensity score overlap weighting (ATO estimand) stratified by heart-failure ejection-fraction class using the WeightIt package [21]. The ATO estimand assigns the greatest weight to patients with a propensity score near 0.5 (indicating a similar probability of receiving either treatment) and downweights those with a very high or very low probability of receiving one treatment. Stratification was necessary for the HF phenotype to ensure covariate balance was achieved within each ejection-fraction category (HFpEF, HFmrEF, and HFrEF). This method assigns greater weight to patients with a high probability of receiving either treatment (DPP-4 inhibitor or non-DPP-4 inhibitor), thereby emphasizing the population in clinical equipoise and improving both precision and internal validity. The propensity model included demographic, clinical, and medication covariates listed in baseline characteristics, including age, sex, body mass index, heart-failure class, comorbidities (e.g., chronic kidney disease, hypertension, DM-related variables, anemia), and concomitant use of agents such as SGLT-2 inhibitors, GLP-1 RA, ACEi/ARB, β-blockers, and insulin.

Treatment exposure was modeled as a baseline fixed effect and not as a time-varying covariate. Changes in DPP-4 inhibitor therapy during follow-up, including discontinuation or switching, were not explicitly modeled to due to the nature of the study design. This approach is consistent with prior real-world cardiovascular safety studies of glucose-lowering therapies and reflects an intention-to-treat analytic framework.

**Figure A1:**
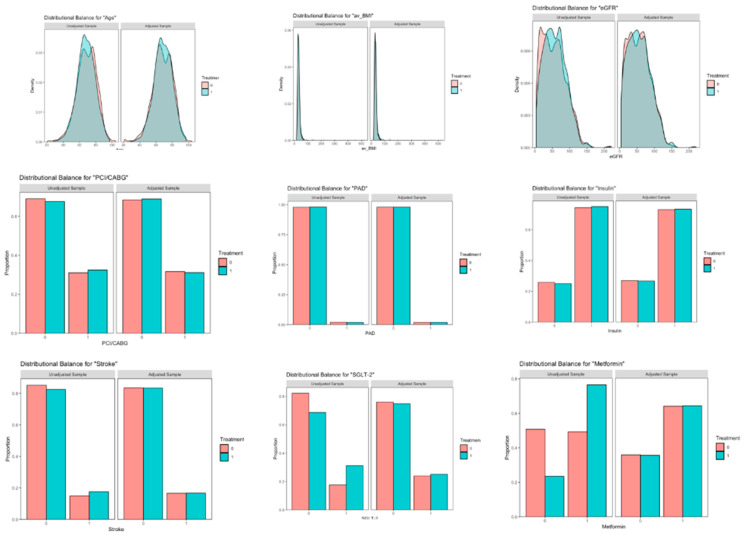

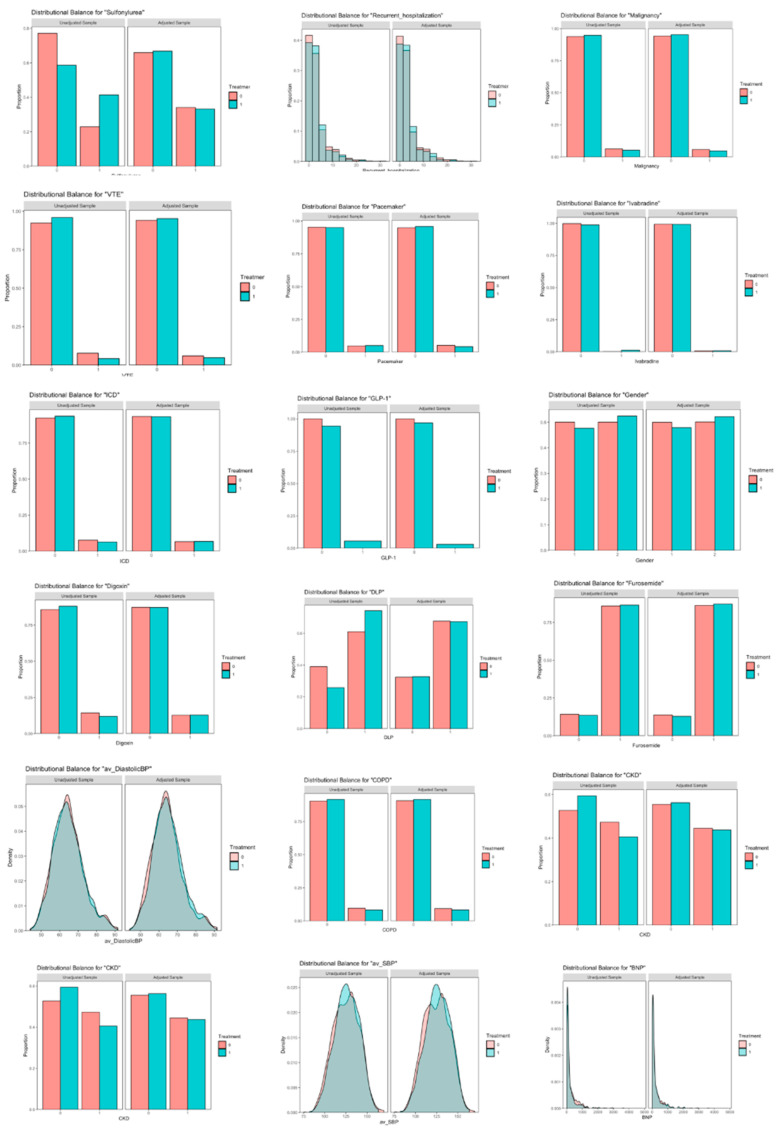

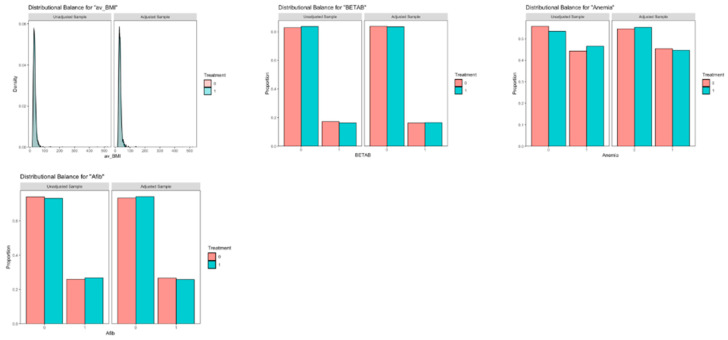
Distributional balance of baseline covariates before and after propensity score overlap weighting. Standardized mean differences are shown for key clinical variables across heart failure phenotypes.

Covariate balance before and after weighting was evaluated using standardized mean differences (SMDs), with |SMD| < 0.2 indicating acceptable balance. Covariate balance was visualized via Love plots generated using the cobalt package (version 4.6.1) in R (version 4.5.2; R Foundation for Statistical Computing, Vienna, Austria). Post-weighting, all covariates demonstrated adequate balance, with only BNP and metformin use remaining marginally imbalanced (|SMD| ≈ 0.20). Substantial improvement in covariate balance was observed after overlap weighting across all HF phenotypes, as illustrated in Figure A1.

The primary outcome was analyzed using weighted logistic regression models to estimate odds ratios (ORs) and 95% confidence intervals (CIs). Interaction terms between DPP-4 inhibitor use and heart-failure ejection-fraction classes (HFpEF, HFmrEF, HFrEF) were included to assess heterogeneity of effect across subtypes. All tests were two-sided, and *p* < 0.05 was considered statistically significant.

All analyses were conducted in R version 4.4.2 using the following packages: MICE (for multiple imputation) [20], WeightIt (for propensity score weighting using the ATO estimand) [21], cobalt (for covariate balance diagnostics and Love plots) [22], tableone (for descriptive summaries) [23], and ggplot2 (for figure generation) [24,25].

## 3. Results

### 3.1. Baseline Characteristics

A total of 3435 patients with type 2 DM and HF were included, of whom 1921 (55.9%) received a DPP-4 inhibitor, and 1514 (44.1%) did not. The distribution of HF phenotypes differed slightly between groups, with HFpEF being the most common (52.6%) followed by HFrEF (35.1%) and HFmrEF (12.3%). Patients in the DPP-4 inhibitor group were marginally younger (median 68 vs. 69 years, *p* = 0.028) and had a higher BMI (31.29 vs. 30.74 kg/m^2^, *p* = 0.015). They also demonstrated significantly lower baseline BNP levels (82.1 vs. 121.5 pg/mL, *p* < 0.001) and higher eGFR (55 vs. 52 mL/min/1.73 m^2^, *p* = 0.007). Comorbidity profiles showed notable differences: DPP-4 inhibitor users had a lower prevalence of CKD (38.4% vs. 45.8%, *p* < 0.001), VTE (4.2% vs. 7.7%, *p* < 0.001), and dyslipidemia was more frequent in the DPP-4 inhibitor group (68.8% vs. 57.5%, *p* < 0.001). Concomitant medication use also varied, with significantly higher rates of SGLT2 inhibitors (31.3% vs. 17.6%), GLP-1 RA (5.6% vs. 0%), metformin (76.5% vs. 49.1%), and sulfonylureas (41.4% vs. 22.7%) in the DPP-4 inhibitor cohort (all *p* < 0.001). Complete baseline data are provided in Table 1.

### 3.2. Baseline Characteristics Stratified by HF Phenotype

Stratification by ejection-fraction class demonstrated apparent demographic and clinical differences across HFpEF, HFmrEF, and HFrEF subtypes. For HFpEF, DPP-4 inhibitor users tended to be younger (71 vs. 74 years) and had lower BNP levels compared with non-users. Similar trends of younger age, lower BNP, and higher eGFR were observed for DPP-4 inhibitor users in HFmrEF and HFrEF groups (all *p* < 0.001). Across all HF phenotypes, DPP-4 inhibitor users had significantly higher use of contemporary cardiometabolic therapies, including SGLT2 inhibitors and GLP-1 RA. Full stratified results are available in Table A1 of Appendix A.

### 3.3. Covariate Balance After Weighting

**Figure 1:**
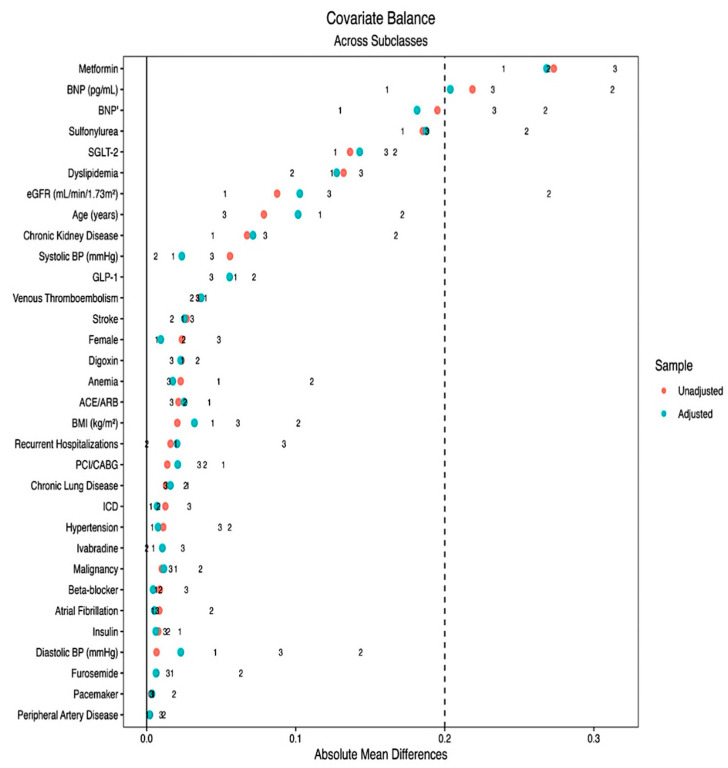
Love plot of the covariate balance before and after weighting across HF Phenotype. Each HF phenotype stratum is presented as number 1 = Preserved ejection fraction; 2 = mildly reduced ejection fraction; 3 = Reduced ejection fraction. Abbreviations: ACEi = angiotensin-converting enzyme inhibitor; ARB = angiotensin II receptor blocker; BMI = body mass index; BNP = B-type natriuretic peptide; BP = blood pressure; CKD = chronic kidney disease; COPD = chronic obstructive pulmonary disease; DLP = dyslipidemia; eGFR = estimated glomerular filtration rate; ICD = implantable cardioverter-defibrillator; PAD = peripheral artery disease; PCI/CABG = percutaneous coronary intervention or coronary artery bypass graft; SGLT-2 = sodium–glucose co-transporter-2; GLP-1 = glucagon-like peptide receptor agonist-1; VTE = venous thromboembolism.

Propensity scores overlap weighting (ATO) achieved substantial improvement in covariate balance across HF phenotypes. After weighting, all standardized mean differences (SMDs) were <0.20, meeting prespecified thresholds for acceptable balance. Most covariates approached near-perfect balance (SMD < 0.05). Only BNP and metformin use remained marginally imbalanced (SMD ≈ 0.20), consistent with their substantial baseline differences. Details are presented in Table 2 and in the Love plot, Figure 1.

Given the marginal residual imbalance observed for baseline BNP and metformin use after overlap weighting (standardized mean difference ≈ 0.20), a sensitivity analysis was performed in which both variables were additionally included as covariates in the weighted regression models. Effect estimates for the association between DPP-4 inhibitor use and cardiovascular mortality remained unchanged, and no meaningful differences were observed compared with the primary weighted analysis. These findings indicate that the observed residual imbalance is unlikely to have materially influenced the primary results.

### 3.4. CV Mortality Outcomes

In the unweighted analyses, use of DPP-4 inhibitors was associated with numerically lower CV mortality across all HF phenotypes, although these differences did not reach statistical significance for patients with HFpEF or HFrEF. Among patients with HFpEF, CV mortality occurred in 10.1% of those receiving DPP-4 inhibitors compared with 13.0% of those not receiving these agents (odds ratio [OR] 0.75, 95% confidence interval [CI] 0.53–1.07; *p* = 0.116). In the HFmrEF subgroup, cardiovascular mortality was observed in 5.1% of patients treated with DPP-4 inhibitors compared with 6.5% of untreated patients. Although the corresponding OR suggested lower odds of cardiovascular death (OR 0.32, 95% CI 0.12–0.85), this estimate was unstable and did not remain statistically significant in tabulated analyses, reflecting limited event counts and residual confounding. Among patients with HFrEF, CV mortality occurred in 7.8% of those receiving DPP-4 inhibitors and 9.1% of those not receiving DPP-4 inhibitors (OR 0.79, 95% CI 0.49–1.28; *p* = 0.335).

**Figure 2:**
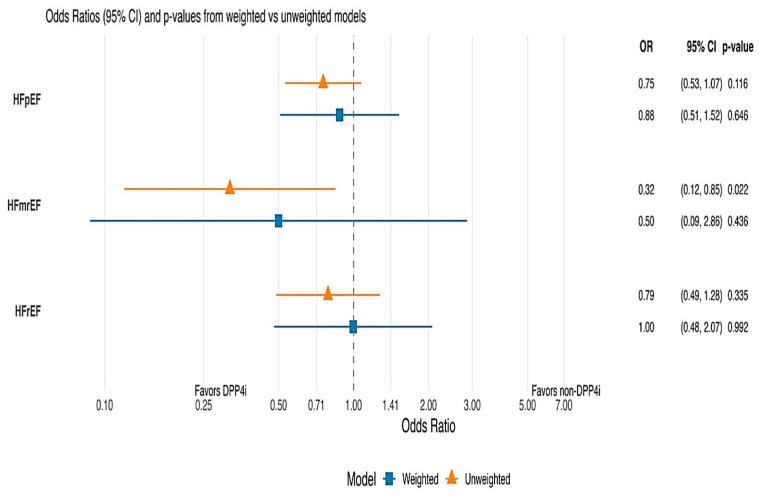
Effect of DPP4i on CV death by HF phenotypes at six months. The unweighted model for CV death by HF phenotype showed that HFmrEF and DPP4i use were associated with statistically significantly lower odds of CV death, but this effect was not observed in the weighted model.

After applying overlap weighting to balance baseline clinical characteristics between treatment groups, differences in CV mortality were further attenuated across all HF phenotypes. In the weighted analysis, CV mortality occurred in 7.1% of patients with HFpEF who were receiving DPP-4 inhibitors, compared with 8.0% of those not receiving these agents (OR 0.88, 95% CI 0.51–1.52; *p* = 0.646). Among patients with HFmrEF, weighted event rates were 2.6% in the DPP-4 inhibitor group and 5.0% in the non–DPP-4 inhibitor group (OR 0.50, 95% CI 0.09–2.86; *p* = 0.436). In patients with HFrEF, CV mortality rates were identical between groups (6.4% in both groups; OR 1.00, 95% CI 0.48–2.07; *p* = 0.992). No significant interaction was observed between DPP-4 inhibitor use and HF phenotype for CV mortality (interaction *p* > 0.05). Detailed results are presented in Table 3 and Figure 2.

## 4. Discussion

In this large real-world cohort of patients with established HF, treatment with DPP-4 inhibitors, predominantly sitagliptin, was not associated with an increased odds of short-term CV mortality after robust adjustment. This neutral association was consistent across HF phenotypes, including HFpEF, HFmrEF, and HFrEF. Although unadjusted analyses suggested lower mortality among patients receiving DPP-4 inhibitors, this apparent benefit was no longer evident after propensity score overlap weighting, underscoring the influence of baseline clinical differences rather than a true treatment effect. Patients treated with DPP-4 inhibitors tended to be younger, had more preserved renal function, lower baseline BNP levels, and were more frequently treated with contemporary cardiometabolic therapies such as metformin, SGLT2 inhibitors, and GLP-1 RA. Together, these characteristics suggest preferential prescribing to patients who were clinically more stable, a pattern consistent with channeling bias. Once these prognostically relevant factors were balanced, DPP-4 inhibitors demonstrated CV neutrality rather than benefit or harm.

Across HF phenotypes, adjusted analyses did not reveal meaningful heterogeneity in the association between DPP-4 inhibitor use and CV mortality. While the HFmrEF subgroup showed a modestly favorable signal in unadjusted analyses, this association was attenuated after weighting and accompanied by wide confidence intervals. HFmrEF represents a remarkably heterogeneous and evolving phenotype, encompassing patients with improving or worsening systolic function and a broad range of underlying etiologies. In addition, the adequate sample size within this subgroup was substantially reduced after overlap weighting, limiting precision and statistical power. Taken together, these factors suggest that the initial HFmrEF signal likely reflects residual confounding and imprecision rather than an actual phenotype-specific effect.

The present findings are consistent with the broader CV outcomes literature, which indicates that the HF safety profile of DPP-4 inhibitors is not uniform across the class and may vary by individual agent. In SAVOR-TIMI 53, saxagliptin was associated with an increased risk of HF hospitalization despite neutral ischemic outcomes, raising early concerns regarding DPP-4 inhibition in patients with HF [26]. In contrast, the TECOS trial demonstrated CV neutrality with sitagliptin, including no excess risk of HF hospitalization, even among participants with pre-existing HF [15]. Similarly, the EXAMINE trial showed no significant increase in HF events with alogliptin following acute coronary syndrome, although estimates in HF subgroups were imprecise [27]. In the VIVIDD trial, vildagliptin did not adversely affect left ventricular ejection fraction in patients with established HFrEF, although increases in left ventricular volumes were observed; the clinical implications of which remain uncertain [13]. Collectively, these data suggest that the HF signal observed with saxagliptin may not represent a class-wide effect but instead reflects agent-specific characteristics or differences in patient selection. Given that sitagliptin accounted for the majority of DPP-4 inhibitor exposure in our cohort, the absence of excess CV mortality across HF phenotypes aligns closely with the neutral safety profile reported in TECOS and supports the CV safety of sitagliptin in routine clinical practice [16].

Within the context of contemporary HF management, the observed neutrality of DPP-4 inhibitor use with respect to short-term CV mortality is clinically reasonable. Prior CV outcome trials established overall CV safety for several DPP-4 inhibitors but were not designed to specifically evaluate mortality within well-characterized HF populations or within modern therapeutic environments [13,15,16,26,27,28]. Our study extends this evidence by demonstrating consistent mortality neutrality across HF phenotypes in a real-world cohort managed with contemporary cardiometabolic therapies. Moreover, CV mortality may be a relatively insensitive endpoint for detecting modest drug-related effects, particularly over a limited follow-up period. In current practice, any small independent impact of DPP-4 inhibition on outcomes is likely overshadowed by the widespread use of therapies with proven mortality benefit. In this cohort, patients receiving DPP-4 inhibitors were more frequently treated with SGLT2 inhibitors and GLP-1 RA, agents that have consistently been shown to reduce HF events and CV death [8,29,30]. Against this background of optimized care, the absence of excess mortality associated with DPP-4 inhibitor use supports their role as cardiovascularly safe adjunctive glucose-lowering therapies rather than modifiers of HF prognosis.

This study has several important strengths. It focuses on a large, real-world population of patients with established HF and DM, a group that has been relatively underrepresented in prior DPP-4 inhibitor trials. HF was rigorously phenotyped rather than treated as a single entity, allowing for a more granular assessment of potential heterogeneity in treatment associations. In addition, the use of propensity score overlap weighting targeting the average treatment effect in the overlap population enabled effective balancing of clinically relevant covariates while maintaining focus on patients for whom clinical equipoise exists. This approach enhances interpretability in routine practice and reduces the influence of extreme propensity scores. Finally, the predominance of sitagliptin exposure enhances clinical relevance and facilitates comparison with randomized trial data supporting its CV safety.

Several limitations warrant consideration. The six-month follow-up period may not fully capture the longer-term effects of DPP-4 inhibitor use on heart failure progression, ventricular remodeling, or recurrent hospitalizations. This timeframe was selected to evaluate short-term cardiovascular safety in routine clinical practice and reflects the available longitudinal data at the time of analysis. The analysis was restricted to CV mortality, and other clinically significant outcomes, such as HF hospitalization, renal function trajectories, or changes in left ventricular function, were not evaluated and may be more sensitive to treatment-related effects.

With respect to external validity, this study reflects real-world practice within a large tertiary academic medical center serving a population with a high burden of cardiometabolic comorbidity. Prescribing patterns, access to contemporary HF therapies, and utilization of DPP-4 inhibitors—particularly the predominance of sitagliptin—may differ across healthcare systems and geographic regions. As such, while the observed cardiovascular safety signal is biologically plausible and consistent with randomized trial data, absolute risk estimates and treatment distributions may vary in settings with different healthcare delivery models, formulary structures, and prescribing preferences.

Although overlap weighting achieved excellent balance across most measured covariates, marginal residual imbalance remained for BNP and metformin, and residual confounding from unmeasured factors remains possible, including frailty, socioeconomic factors, medication adherence, duration and severity of diabetes, dosing or renal adjustment of DPP-4 inhibitors, and distinctions between new and prevalent users. However, sensitivity analyses adjusting for both BNP and metformin yielded consistent results, supporting the robustness of the primary findings. Finally, while the results primarily reflect the safety profile of sitagliptin, heterogeneity in cardiovascular signals across DPP-4 inhibitors observed in prior trials suggests that agent-specific effects cannot be excluded. Future studies with longer follow-up, broader outcome assessment, and drug-level analyses will be necessary to further define the role of individual DPP-4 inhibitors in contemporary heart failure care.

## 5. Conclusions

In this large, real-world cohort of patients with established HF and DM, DPP-4 inhibitor use, primarily sitagliptin, was not associated with increased odds of short-term CV death. After thorough adjustment using propensity score overlap weighting, this neutral association remained consistent across different HF types, with no evidence of varied effects in HFpEF, HFmrEF, or HFrEF. Although unadjusted analyses indicated lower mortality among DPP-4 inhibitor users, this difference was attributable to baseline clinical differences, underscoring the need to account for confounding factors in observational studies. Overall, these findings support the CV safety of DPP-4 inhibitors in patients with established HF when used alongside current cardiometabolic treatment plans, reinforcing their role as additional glucose-lowering agents rather than as modifiers of HF prognosis. Future research with longer follow-up periods and broader outcome assessments is necessary to better understand agent-specific effects and long-term impacts in this high-risk group.

## Figures and Tables

**Table 1:** Baseline characteristics.

Variable	Overall (N = 3435)	Non-DPP-4 Inhibitor (N = 1514)	DPP-4 Inhibitor (N = 1921)	*p*-Value
HF phenotype, n (%)				0.083
HFpEF	1807 (52.6)	767 (50.7)	1040 (54.1)	
HFmrEF	421 (12.3)	185 (12.2)	236 (12.3)	
HFrEF	1207 (35.1)	562 (37.1)	645 (33.6)	
Age (years, median [25–75th])	69 [61–77]	69 [61–77.75]	68 [61–76]	0.028
Female gender (%)	1763 (51.3)	757 (50.0)	1006 (52.4)	0.179
BMI (kg/m^2^, median [25–75th])	31.03 [26.48–36.19]	30.74 [26.23–36.06]	31.29 [26.83–36.29]	0.015
Recurrent hospitalizations (median [IQR])	2 [1–4]	2 [1–4]	2 [1–4]	0.903
Systolic BP (mmHg, median [25–75th])	125.63 [114.77–136.20]	124.95 [113.35–135.77]	126.05 [115.66–136.48]	0.102
Diastolic BP (mmHg, median [25–75th])	63.67 [58.58–68.99]	63.62 [58.48–69.02]	63.70 [58.72–68.97]	0.976
BNP (pg/mL, median [IQR])	95.8 [33.7–280.4]	121.5 [40.9–377.1]	82.1 [31.1–222.2]	<0.001
eGFR (mL/min/1.73 m^2^, median [IQR])	54 [31–78]	52 [27–76.5]	55 [34–78]	0.007
Past medical history, n (%)				
VTE	197 (5.7)	117 (7.7)	80 (4.2)	<0.001
Hypertension	2993 (87.1)	1331 (87.9)	1662 (86.5)	0.192
Dyslipidemia	2192 (63.8)	870 (57.5)	1322 (68.8)	<0.001
Chronic kidney disease	1430 (41.6)	693 (45.8)	737 (38.4)	<0.001
Stroke	537 (15.6)	217 (14.3)	320 (16.7)	0.027
PAD	61 (1.8)	29 (1.9)	32 (1.7)	0.762
Anemia	1461 (42.5)	635 (41.9)	826 (43.0)	0.182
COPD	285 (8.3)	138 (9.1)	147 (7.7)	0.224
Malignancy	188 (5.5)	91 (6.0)	97 (5.0)	0.343
Afib	843 (24.5)	375 (24.8)	468 (24.4)	0.848
PCI/CABG	1036 (30.2)	452 (29.9)	584 (30.4)	0.363
Pacemaker	155 (4.5)	69 (4.6)	86 (4.5)	1.000
ICD	227 (6.6)	111 (7.3)	116 (6.0)	0.227
Medication use history, n (%)				
β-blocker	573 (16.7)	260 (17.2)	313 (16.3)	0.522
ACEi or ARB	1121 (32.6)	476 (31.4)	645 (33.6)	0.197
Digoxin	445 (13.0)	216 (14.3)	229 (11.9)	0.048
Furosemide	2962 (86.2)	1300 (85.9)	1662 (86.5)	0.616
Ivabradine	27 (0.8)	3 (0.2)	24 (1.2)	0.001
SGLT-2 inhibitor	868 (25.3)	267 (17.6)	601 (31.3)	<0.001
GLP-1 RA	107 (3.1)	0 (0.0)	107 (5.6)	<0.001
Metformin	2214 (64.5)	744 (49.1)	1470 (76.5)	<0.001
Sulfonylurea	1139 (33.2)	344 (22.7)	795 (41.4)	<0.001
Insulin	2556 (74.4)	1117 (73.8)	1439 (74.9)	0.568

Missing data (overall n = 3435): VTE = 7 (0.2%), Stroke = 198 (5.8%), PAD = 199 (5.8%), Anemia = 206 (6.0%), COPD = 198 (5.8%), Malignancy = 196 (5.7%), Afib = 198 (5.8%), PCI/CABG = 201 (5.9%), Pacemaker = 202 (5.9%), ICD = 201 (5.9%), Metformin = 2 (0.1%), Sulfonylurea = 7 (0.2%), Insulin = 8 (0.2%). Tests used: Mann–Whitney U test for continuous non-normally distributed variables; Chi-square test for categorical variables. Abbreviations: ACEi = angiotensin-converting enzyme inhibitor; ARB = angiotensin II receptor blocker; Afib = atrial fibrillation; BMI = body mass index; BNP = B-type natriuretic peptide; BP = blood pressure; COPD = chronic obstructive pulmonary disease; eGFR = estimated glomerular filtration rate; HF = heart failure; HFpEF = HF with preserved EF; HFmrEF = HF with mildly reduced EF; HFrEF = HF with reduced EF; ICD = implantable cardioverter-defibrillator; PAD = peripheral artery disease; PCI/CABG = percutaneous coronary intervention or coronary artery bypass graft; SGLT-2 = sodium-glucose co-transporter-2; GLP-1 RA = glucagon-like peptide-1receptor agonist; VTE = venous thromboembolism.

**Table 2:** Averaged Covariate Balance Before and After Weighting Across HF Phenotypes (Non-DPP4i vs. DPP4i).

Variable	Mean (SD) Unadjusted/or %	Unadjusted Absolute Difference (SMD)	Adjusted Mean (SD) or %	Adjusted Absolute Difference (SMD)
Age (years)	68.93 (12.02) vs. 68.07 (10.94)	0.0787	69.18 (11.96) vs. 68.07 (10.94)	0.1017
Female gender (%)	50.0 vs. 52.4	0.0237	51 vs. 52	0.0095
BMI (kg/m^2^)	32.86 (23.89) vs. 32.59 (13.27)	0.0206	33.01 (23.51) vs. 32.59 (13.27)	0.0321
Recurrent hospitalizations	3.41 (3.82) vs. 3.36 (3.65)	0.0161	3.43 (3.83) vs. 3.36 (3.65)	0.0204
Systolic BP (mmHg)	125.34 (15.22) vs. 126.14 (14.28)	0.0559	125.80 (15.16) vs. 126.14 (14.28)	0.0236
Diastolic BP (mmHg)	64.60 (8.17) vs. 64.66 (8.11)	0.0067	64.47 (8.14) vs. 64.66 (8.11)	0.0229
VTE (%)	7.7 vs. 4.2	0.0356	7.8 vs. 4.2	0.0366
Hypertension (%)	91.6 vs. 92.7	0.0111	91.9 vs. 92.7	0.0077
DLP (%)	60.9 vs. 74.2	0.1322	61.4 vs. 74.2	0.1276
CKD (%)	47.3 vs. 40.6	0.0674	47.7 vs. 40.6	0.0714
Stroke (%)	14.9 vs. 17.5	0.0268	15.0 vs. 17.5	0.0254
PAD (%)	2.1 vs. 1.8	0.0023	2.0 vs. 1.8	0.0020
Anemia (%)	44.3 vs. 46.5	0.0228	44.8 vs. 46.5	0.0175
COPD (%)	9.6 vs. 8.3	0.0130	9.9 vs. 8.3	0.0160
Malignancy (%)	6.3 vs. 5.3	0.0102	6.4 vs. 5.3	0.0116
Afib (%)	26.0 vs. 26.8	0.0085	26.3 vs. 26.8	0.0052
PCI/CABG (%)	31.0 vs. 32.4	0.0139	30.3 vs. 32.4	0.0209
Pacemaker (%)	4.8 vs. 5.1	0.0029	4.7 vs. 5.1	0.0036
ICD (%)	7.7 vs. 6.4	0.0126	7.1 vs. 6.4	0.0071
BNP (pg/mL)	287.7 (470.4) vs. 208.4 (362.5)	0.2186	282.3 (463.4) vs. 208.4 (362.5)	0.2038
BNP′(pg/mL)	59.61 (150.63) vs. 37.82 (111.64)	0.1951	58.08 (148.09) vs. 37.82 (111.64)	0.1815
eGFR (mL/min/1.73 m^2^)	55.30 (34.04) vs. 58.04 (31.28)	0.0875	54.82 (33.84) vs. 58.04 (31.28)	0.1028
β-blocker (any)	17.2 vs. 16.3	0.0088	16.7 vs. 16.3	0.0044
ACEi/ARB (%)	31.4 vs. 33.6	0.0214	31.1 vs. 33.6	0.0252
Digoxin (%)	14.3 vs. 11.9	0.0235	14.2 vs. 11.9	0.0229
Furosemide (%)	85.9 vs. 86.5	0.0065	85.9 vs. 86.5	0.0063
Ivabradine (%)	0.2 vs. 1.3	0.0105	0.2 vs. 1.3	0.0106
SGLT-2 inhibitor (%)	17.6 vs. 31.3	0.1365	17.0 vs. 31.3	0.1430
GLP-1 RA (%)	0.0 vs. 5.6	0.0557	0.0 vs. 5.6	0.0557
Metformin (%)	49.2 vs. 76.5	0.2732	49.7 vs. 76.5	0.2684
Sulfonylurea (%)	22.9 vs. 41.4	0.1853	22.6 vs. 41.4	0.1875
Insulin (%)	74.2 vs. 74.9	0.0079	74.3 vs. 74.9	0.0062

Mean (SD) values are shown for continuous variables, proportions for binary variables. Unadjusted and adjusted absolute differences represent standardized mean differences (SMD) before and after weighting. Balance status: Balanced if |SMD| < 0.2, Not balanced if |SMD| ≥ 0.2. Only BNP and Metformin remained marginally unbalanced after weighting. Note: BNP was modeled with restricted cubic splines (rcs) with 3 knots. Balance was achieved using the Average Treatment Effect in the Overlap (ATO) approach, which emphasizes patients with similar probabilities of receiving either treatment, enhancing precision and reducing bias (Greifer & Stuart, 2021) [26]. Multiple imputation was performed prior to weighting to handle missing data using MICE package [20]. One complete imputed dataset was then selected to display covariate balance. Weighting was implemented using the WeightIt package (R) [21] with the ATO estimand, and balance diagnostics were generated using the cobalt package [22]. Abbreviations: ACEi = angiotensin-converting enzyme inhibitor; ARB = angiotensin II receptor blocker; Afib = atrial fibrillation; BMI = body mass index; BNP = B-type natriuretic peptide; BP = blood pressure; CKD = chronic kidney disease; COPD = chronic obstructive pulmonary disease; DLP = dyslipidemia; eGFR = estimated glomerular filtration rate; ICD = implantable cardioverter-defibrillator; PAD = peripheral artery disease; PCI/CABG = percutaneous coronary intervention or coronary artery bypass graft; SGLT-2 = sodium–glucose co-transporter-2; GLP-1 RA = glucagon-like peptide-1 receptor agonist; VTE = venous thromboembolism.

**Table 3:** CV Death Outcomes results.

Unweighted Raw Data	Non-DPP-4 Inhibitor & HFpEF (n = 767)	DPP-4 Inhibitor & HFpEF (n = 1040)	Non-DPP-4 Inhibitor & HFmrEF (n = 185)	DPP-4 Inhibitor & HFmrEF (n = 236)	Non-DPP-4 Inhibitor & HFrEF (n = 562)	DPP-4 Inhibitor & HFrEF (n = 645)
CV death, %	100 (13.0)	105 (10.1)	12 (6.5)	12 (5.1)	51 (9.1)	50 (7.8)
OR (95% CI, *p* value)	0.75 (0.53–1.07, *p* = 0.116)		0.32 (0.12–0.85, *p* = 0.436)		0.79 (0.49–1.28, *p* = 0.335)	
Weighted data	Non-DPP-4 inhibitor & HFpEF (effective sample size = 365)	DPP-4 inhibitor & HFpEF (effective sample size = 365)	Non-DPP-4 inhibitor & HFmrEF (effective sample size = 76)	DPP-4 inhibitor & HFmrEF (effective sample size = 76)	Non-DPP-4 inhibitor & HFrEF (effective sample size = 240)	DPP-4 inhibitor HFrEF (effective sample size = 240)
CV death (weighted data)	29 (8.0)	26 (7.1)	4 (5.0)	2.0 (2.6)	15 (6.4)	15 (6.4)
OR (95% CI, *p* value)	0.88 (0.51–1.52, *p* = 0.646)		0.50 (0.09–2.86, *p* = 0.436)		1.00 (0.48–2.07, *p* = 0.992)	

Balance was achieved using the Average Treatment Effect in the Overlap (ATO) approach, which focuses on patients with similar probabilities of receiving either treatment, thereby enhancing precision and reducing bias. Odds ratios were estimated using weighted logistic regression with a robust variance estimator by interacting the HF phenotype with DPP-4 inhibitor use. Abbreviations: Heart failure with preserved EF (HFpEF) = EF ≥ 50%; Heart failure with mildly reduced EF (HFmrEF) = EF 41–49%; Heart failure with reduced EF (HFrEF) = EF ≤ 40%. OR = odds ratio; CI = confidence interval.

## Data Availability

The data that support the findings of this study are available from the corresponding author upon reasonable request. The availability of these data is restricted by institutional policies and patient confidentiality.

## Abbreviations

The following abbreviations are used in this manuscript:

ACS	Acute coronary syndrome
ATO	Average treatment effect in the overlap population
BMI	Body mass index
CI	Confidence interval
CKD	Chronic kidney disease
CV	Cardiovascular
DPP-4i	Dipeptidyl peptidase-4 inhibitor
HF	Heart failure
HFmrEF	Heart failure with mildly reduced ejection fraction
HFpEF	Heart failure with preserved ejection fraction
HFrEF	Heart failure with reduced ejection fraction
ICD	International Classification of Diseases
IRB	Institutional Review Board
MI	Myocardial infarction
OR	Odds ratio
PS	Propensity score
SD	Standard deviation

## Appendix A

**Table A1:** Baseline characteristics stratified by DPP4i use and heart failure ejection fraction class in the pre-propensity score overlap weighting sample (raw data).

Variable	Non-DPP-4i & HFpEF (n = 767)	DPP-4i & HFpEF (n = 1040)	Non-DPP-4 & HFmEF (n = 185)	DPP-4i & HFmEF (n = 236)	Non-DPP-4i & HFrEF (n = 562)	DPP-4 HFrEF (n = 645)	*p*-Value
Age (years, median [25–75th])	74 [65–80]	71 [65–78]	68 [61–76]	66 [60–73]	65 [57–74]	64 [57–72]	<0.001
Female gender (%)	520 (67.8)	698 (67.1)	83 (44.9)	100 (42.4)	154 (27.4)	208 (32.2)	<0.001
BMI (kg/m^2^, median [25–75th])	32.99 [28.25–38.31]	33.06 [28.47–38.59]	29.11 [25.66–34.57]	32.81 [27.52–37.32]	28.20 [24.81–32.02]	28.25 [24.62–32.72]	<0.001
Recurrent hospitalizations (median [IQR])	2 [1–5]	2 [1–5]	2 [1–4]	3 [1–5]	2 [1–4]	2 [1–3]	<0.001
Systolic BP (mmHg)	130.7 [119.5–140.6]	131.2 [121.9–140.2]	129.0 [117.6–137.6]	126.7 [118.7–135.3]	116.2 [106.5–126.8]	117.9 [107.6–125.3]	<0.001
Diastolic BP (mmHg)	61.9 [57.0–66.8]	61.4 [56.9–66.6]	64.4 [59.2–69.4]	65.0 [60.2–69.1]	65.8 [60.9–70.9]	66.6 [61.5–71.8]	<0.001
BNP (pg/mL, median [IQR])	84.3 [35.4–240.8]	64.9 [29.1–145.1]	160.3 [57.4–464.1]	62.0 [17.0–202.4]	179.7 [48.0–564.8]	124.6 [42.8–428.8]	<0.001
eGFR (mL/min/1.73 m^2^)	46 [25–74]	49 [31–73]	57 [21.5–80.5]	68 [47–86.3]	54 [31–79]	62 [37.5–84]	<0.001
Past medical history, n(%)							
VTE	68 (8.9)	51 (4.9)	15 (8.1)	12 (5.1)	34 (6.0)	17 (2.6)	<0.001
Hypertension	695 (90.6)	914 (87.9)	165 (89.2)	191 (80.9)	471 (83.8)	557 (86.4)	<0.001
Dyslipidemia	473 (61.7)	750 (72.1)	111 (60.0)	159 (67.4)	286 (50.9)	413 (64.0)	<0.001
Chronic kidney disease	378 (49.3)	460 (44.2)	94 (50.8)	79 (33.5)	221 (39.3)	198 (30.7)	<0.001
Stroke	121 (15.8)	186 (17.9)	28 (15.1)	38 (16.1)	68 (12.1)	96 (14.9)	0.022
PAD	15 (2.0)	18 (1.7)	1 (0.5)	4 (1.7)	13 (2.3)	10 (1.6)	0.744
Anemia	364 (47.5)	526 (50.6)	80 (43.2)	74 (31.4)	191 (34.0)	226 (35.0)	<0.001
COPD	98 (12.8)	103 (9.9)	14 (7.6)	22 (9.3)	26 (4.6)	22 (3.4)	<0.001
Malignancy	59 (7.7)	62 (6.0)	9 (4.9)	19 (8.1)	23 (4.1)	16 (2.5)	<0.001
Atrial fibrillation	223 (29.1)	294 (28.3)	37 (20.0)	57 (24.2)	115 (20.5)	117 (18.1)	<0.001
PCI/CABG	158 (20.6)	262 (25.2)	68 (36.8)	88 (37.3)	226 (40.2)	234 (36.3)	<0.001
Pacemaker	31 (4.0)	44 (4.2)	6 (3.2)	10 (4.2)	32 (5.7)	32 (5.0)	0.707
ICD	14 (1.8)	18 (1.7)	3 (1.6)	7 (3.0)	94 (16.7)	91 (14.1)	<0.001
Medication use history, n (%)							
β-blocker	91 (11.9)	130 (12.5)	32 (17.3)	43 (18.2)	137 (24.4)	140 (21.7)	<0.001
ACEi or ARB	203 (26.5)	319 (30.7)	62 (33.5)	73 (30.9)	211 (37.5)	253 (39.2)	<0.001
Digoxin	107 (14.0)	120 (11.5)	22 (11.9)	20 (8.5)	87 (15.5)	89 (13.8)	0.062
Furosemide	658 (85.8)	910 (87.5)	163 (88.1)	193 (81.8)	479 (85.2)	559 (86.7)	0.252
Ivabradine	1 (0.1)	6 (0.6)	0 (0.0)	0 (0.0)	2 (0.4)	18 (2.8)	<0.001
SGLT-2 inhibitor	74 (9.6)	232 (22.3)	35 (18.9)	84 (35.6)	158 (28.1)	285 (44.2)	<0.001
GLP-1 agonist	0 (0.0)	62 (6.0)	0 (0.0)	17 (7.2)	0 (0.0)	28 (4.3)	<0.001
Metformin	421 (54.9)	820 (78.8)	92 (49.7)	181 (76.7)	231 (41.1)	469 (72.7)	<0.001
Sulfonylurea	160 (20.9)	397 (38.2)	32 (17.3)	101 (42.8)	152 (27.0)	297 (46.0)	<0.001
Insulin	578 (75.4)	811 (78.0)	143 (77.3)	179 (75.8)	396 (70.5)	449 (69.6)	0.002

Missing data (overall n = 3435): VTE = 7 (0.2%), HTN = 195 (5.7%), DLP = 208 (6.1%), CKD = 200 (5.8%), Stroke = 198 (5.8%), PAD = 199 (5.8%), Anemia = 206 (6.0%), COPD = 198 (5.8%), Malignancy = 196 (5.7%), Afib = 198 (5.8%), PCI/CABG = 201 (5.9%), Pacemaker = 202 (5.9%), ICD = 201 (5.9%), Metformin = 2 (0.1%), Sulfonylurea = 7 (0.2%), Insulin = 8 (0.2%). Tests used: Kruskal–Wallis test for continuous non-normally distributed variables; Chi-square test for categorical variables. Abbreviations: ACEi = angiotensin-converting enzyme inhibitor; ARB = angiotensin II receptor blocker; Afib = atrial fibrillation; BMI = body mass index; BNP = B-type natriuretic peptide; BP = blood pressure; CKD = chronic kidney disease; COPD = chronic obstructive pulmonary disease; DLP = dyslipidemia; eGFR = estimated glomerular filtration rate; HF = heart failure; HFpEF = HF with preserved EF; HFmEF = HF with mildly reduced EF; HFrEF = HF with reduced EF; ICD = implantable cardioverter-defibrillator; PAD = peripheral artery disease; PCI/CABG = percutaneous coronary intervention or coronary artery bypass graft; SGLT-2 = sodium–glucose co-transporter-2; GLP-1 = glucagon-like peptide-1; VTE = venous thromboembolism.

## References

[1] International Diabetes Federation (2021). IDF Diabetes Atlas.

[2] Jia G., Hill M.A., Sowers J.R. (2018). Diabetic cardiomyopathy: An update of mechanisms contributing to this clinical entity. Circ. Res..

[3] Kannel W.B., McGee D.L. (1979). Diabetes and cardiovascular disease. The Framingham Study. JAMA.

[4] Nichols G.A., Hillier T.A., Erbey J.R., Brown J.B. (2001). Congestive heart failure in type 2 diabetes: Prevalence, incidence, and risk factors. Diabetes Care.

[5] Kenny H.C., Abel E.D. (2019). Heart failure in type 2 diabetes mellitus. Circ. Res..

[6] Benjamin E.J., Muntner P., Alonso A., Bittencourt M.S., Callaway C.W., Carson A.P., Chamberlain A.M., Chang A.R., Cheng S., Das S.R. (2019). Heart disease and stroke statistics—2019 update: A report from the American Heart Association. Circulation.

[7] McDonagh T.A., Metra M., Adamo M., Gardner R.S., Baumbach A., Böhm M., Burri H., Butler J., Čelutkienė J., Chioncel O. (2021). ESC Guidelines for the diagnosis and treatment of acute and chronic heart failure. Eur. Heart J..

[8] Zinman B., Wanner C., Lachin J.M., Fitchett D., Bluhmki E., Hantel S., Mattheus M., Devins T., Johansen O.E., Woerle H.J. (2015). Empagliflozin, cardiovascular outcomes, and mortality in type 2 diabetes. N. Engl. J. Med..

[9] Marso S.P., Daniels G.H., Brown-Frandsen K., Kristensen P., Mann J.F.E., Nauck M.A., Nissen S.E., Pocock S., Poulter N.R., Ravn L.S. (2016). Liraglutide and cardiovascular outcomes in type 2 diabetes. N. Engl. J. Med..

[10] Deacon C.F. (2011). Dipeptidyl peptidase-4 inhibitors in the treatment of type 2 diabetes: A comparative review. Diabetes Obes. Metab..

[11] Drucker D.J., Nauck M.A. (2006). The incretin system: Glucagon-like peptide-1 receptor agonists and dipeptidyl peptidase-4 inhibitors in type 2 diabetes. Lancet.

[12] Mulvihill E.E., Drucker D.J. (2014). Pharmacology, physiology, and mechanisms of action of dipeptidyl peptidase-4 inhibitors. Endocr. Rev..

[13] Green J.B., Bethel M.A., Armstrong P.W., Buse J.B., Engel S.S., Garg J., Josse R., Kaufman K.D., Koglin J., Korn S. (2015). Effect of sitagliptin on cardiovascular outcomes in type 2 diabetes. N. Engl. J. Med..

[14] Rosenstock J., Perkovic V., Johansen O.E., Cooper M.E., Kahn S.E., Marx N., Alexander J.H., Pencina M., Toto R.D., Wanner C. (2019). Effect of linagliptin vs placebo on cardiovascular and kidney outcomes in type 2 diabetes (CARMELINA). N. Engl. J. Med..

[15] Scirica B.M., Bhatt D.L., Braunwald E., Steg P.G., Davidson J., Hirshberg B., Ohman P., Frederich R., Wiviott S.D., Hoffman E.B. (2013). Saxagliptin and cardiovascular outcomes in patients with type 2 diabetes mellitus (SAVOR-TIMI 53). N. Engl. J. Med..

[16] White W.B., Cannon C.P., Heller S.R., Nissen S.E., Bergenstal R.M., Bakris G.L., Perez A.T., Fleck P.R., Mehta C.R., Kupfer S. (2013). Alogliptin after acute coronary syndrome in patients with type 2 diabetes (EXAMINE). N. Engl. J. Med..

[17] Kosiborod M., Cavender M.A., Fu A.Z., Wilding J.P., Khunti K., Holl R.W., Norhammar A., Birkeland K.I., Jørgensen M.E., Thuresson M. (2019). Lower risk of heart failure and death in patients initiated on SGLT2 inhibitors vs. DPP-4 inhibitors: Multinational cohort study. BMJ.

[18] Udell J.A., Cavender M.A., Bhatt D.L., Chatterjee S., Farkouh M.E., Scirica B.M. (2015). Glucose-lowering drugs or strategies and cardiovascular outcomes in patients with or at risk for type 2 diabetes: A meta-analysis of randomised controlled trials. Lancet Diabetes Endocrinol..

[19] Savarese G., Perrone-Filardi P., D’Amore C., Vitale C., Trimarco B., Pani L., Rosano G.M. (2015). Cardiovascular effects of dipeptidyl peptidase-4 inhibitors in diabetic patients: A meta-analysis. Int. J. Cardiol..

[20] Aljefree N., Ahmed F. (2015). Prevalence of cardiovascular disease and associated risk factors among Saudi population: Review. Int. J. Health Sci..

[21] van Buuren S., Groothuis-Oudshoorn K. (2011). Mice: Multivariate imputation by chained equations in R. J. Stat. Softw..

[22] Greifer N. (2024). WeightIt: Weighting Approaches for Causal Inference. R Package Version 0.14.2. https://CRAN.R-project.org/package=WeightIt.

[23] Greifer N. (2024). Cobalt: Covariate Balance Tables and Plots. R Package Version 4.5.5. https://CRAN.R-project.org/package=cobalt.

[24] Yoshida K., Bartel A. (2024). Tableone: Create “Table 1” to Describe Baseline Characteristics. R Package Version 0.13.2. https://CRAN.R-project.org/package=tableone.

[25] Wickham H. (2016). Ggplot2: Elegant Graphics for Data Analysis.

[26] Greifer N. (2021). Weighting for covariate balance in observational studies. arXiv.

[27] Scirica B.M., Braunwald E., Raz I., Cavender M.A., Morrow D.A., Jarolim P., Udell J.A., Mosenzon O., Im K., Umez-Eronini A.A. (2014). Heart failure, saxagliptin, and diabetes mellitus: Observations from the SAVOR-TIMI 53 randomized trial. Circulation.

[28] McMurray J.J.V., Ponikowski P., Bolli G.B., Lukashevich V., Kozlovski P., Kothny W., Lewsey J.D., Krum H. (2018). Effects of vildagliptin on ventricular function in patients with type 2 diabetes mellitus and heart failure: A randomized placebo-controlled trial (VIVIDD). Eur. J. Heart Fail..

[29] McMurray J.J.V., Solomon S.D., Inzucchi S.E., Køber L., Kosiborod M.N., Martinez F.A., Ponikowski P., Sabatine M.S., Anand I.S., Bělohlávek J. (2019). Dapagliflozin in patients with heart failure and reduced ejection fraction. N. Engl. J. Med..

[30] Anker S.D., Butler J., Filippatos G., Ferreira J.P., Bocchi E., Böhm M., Rocca H.-P.B.-L., Choi D.-J., Chopra V., Chuquiure-Valenzuela E. (2021). Empagliflozin in heart failure with a preserved ejection fraction. N. Engl. J. Med..

